# Insight into pathogenomics and phylogeography of hypervirulent and highly-lethal *Mycobacterium tuberculosis* strain cluster

**DOI:** 10.1186/s12879-023-08413-7

**Published:** 2023-06-23

**Authors:** Igor Mokrousov, Anna Vyazovaya, Egor Shitikov, Maria Badleeva, Olesya Belopolskaya, Dmitry Bespiatykh, Alena Gerasimova, Panayotis Ioannidis, Weiwei Jiao, Polina Khromova, Aleksey Masharsky, Dinara Naizabayeva, Dimitrios Papaventsis, Oksana Pasechnik, João Perdigão, Nalin Rastogi, Adong Shen, Viacheslav Sinkov, Yuriy Skiba, Natalia Solovieva, Silva Tafaj, Violeta Valcheva, Irina Kostyukova, Svetlana Zhdanova, Viacheslav Zhuravlev, Oleg Ogarkov

**Affiliations:** 1 Laboratory of Molecular Epidemiology and Evolutionary Genetics, St. Petersburg Pasteur Institute, St. Petersburg, Russia; 2 Henan International Joint Laboratory of Children’s Infectious Diseases, Henan Children’s Hospital, Children’s Hospital, Zhengzhou University, Zhengzhou Children’s Hospital, Zhengzhou, China; 3 Department of Biomedicine and Genomics, Lopukhin Federal Research and Clinical Center of Physical-Chemical Medicine of Federal Medical Biological Agency, Moscow, 119435 Russia; 4 Department of Infectious Diseases, Dorji Banzarov Buryat State University, Ulan-Ude, Buryatia, Russia; 5 Resource Center Bio-bank Center, Research Park of St. Petersburg State University, St. Petersburg, Russia; 6 Laboratory of Genogeography, Vavilov Institute of General Genetics Russian Academy of Sciences Moscow, Moscow, Russia; 7 National Reference Laboratory for Mycobacteria, Sotiria Chest Diseases Hospital, Athens, Greece; 8 National Clinical Research Center for Respiratory Diseases, Beijing Key Laboratory of Pediatric Respiratory Infection Disease, Beijing Children’s Hospital, Beijing Pediatric Research Institute, Capital Medical University, National Center for Children’s Health, Beijing, China; 9 Department of Epidemiology and Microbiology, Scientific Centre of the Family Health and Human Reproduction Problems, Irkutsk, Russia; 10 Laboratory of Molecular Biology, Almaty Branch of National Center for Biotechnology in Central Reference Laboratory, Almaty, Kazakhstan; 11 Department of Biotechnology, Al-Farabi Kazakh National University, Almaty, Kazakhstan; 12 Department of Public Health, Omsk State Medical University, Omsk, Russia; 13 iMed.ULisboa – Instituto de Investigação do Medicamento, Faculdade de Farmácia, Universidade de Lisboa, Lisbon, Portugal; 14 WHO Supranational TB Reference Laboratory, Unité de la Tuberculose et des Mycobactéries, Institut Pasteur de la Guadeloupe, Abymes, Guadeloupe, France; 15 Henan Children’s Hospital, Children’s Hospital Affiliated to Zhengzhou University, Zhengzhou Children’s Hospital, Zhengzhou, China; 16 St. Petersburg Research Institute of Phthisiopulmonology, St. Petersburg, Russia; 17 National Mycobacteria Reference Laboratory, University Hospital Shefqet Ndroqi, Tirana, Albania; 18 Laboratory of Molecular Genetics of Mycobacteria, The Stephan Angeloff Institute of Microbiology, Bulgarian Academy of Sciences, Sofia, Bulgaria; 19 Bacteriology laboratory, Clinical Tuberculosis Dispensary, Omsk, Russia

**Keywords:** Mycobacterium tuberculosis, Whole genome sequencing, Virulence, Drug resistance, Beijing genotype

## Abstract

**Background:**

. The *Mycobacterium tuberculosis* Beijing genotype is globally spread lineage with important medical properties that however vary among its subtypes. *M. tuberculosis* Beijing 14717-15-cluster was recently discovered as both multidrug-resistant, hypervirulent, and highly-lethal strain circulating in the Far Eastern region of Russia. Here, we aimed to analyze its pathogenomic features and phylogeographic pattern.

**Results:**

. The study collection included *M. tuberculosis* DNA collected between 1996 and 2020 in different world regions. The bacterial DNA was subjected to genotyping and whole genome sequencing followed by bioinformatics and phylogenetic analysis. The PCR-based assay to detect specific SNPs of the Beijing 14717-15-cluster was developed and used for its screening in the global collections. Phylogenomic and phylogeographic analysis confirmed endemic prevalence of the Beijing 14717-15-cluster in the Asian part of Russia, and distant common ancestor with isolates from Korea (> 115 SNPs). The Beijing 14717-15-cluster isolates had two common resistance mutations RpsL Lys88Arg and KatG Ser315Thr and belonged to spoligotype SIT269. The Russian isolates of this cluster were from the Asian Russia while 4 isolates were from the Netherlands and Spain. The cluster-specific SNPs that significantly affect the protein function were identified *in silico* in genes within different categories (lipid metabolism, regulatory proteins, intermediary metabolism and respiration, PE/PPE, cell wall and cell processes).

**Conclusions:**

. We developed a simple method based on real-time PCR to detect clinically significant MDR and hypervirulent Beijing 14717-15-cluster. Most of the identified cluster-specific mutations were previously unreported and could potentially be associated with increased pathogenic properties of this hypervirulent *M. tuberculosis* strain. Further experimental study to assess the pathobiological role of these mutations is warranted.

**Supplementary Information:**

The online version contains supplementary material available at 10.1186/s12879-023-08413-7.

## Introduction

A study of molecular epidemiology and evolution of *Mycobacterium tuberculosis* has been greatly facilitated by the lack of horizontal gene transfer and strictly clonal population structure of this medically relevant biological species. The clonality implies that the population structure is hierarchical and, as we know, consists of large phylogenetic lineages, smaller genetic families or sublineages, and finally clonal clusters. Pathogenetically significant properties may be featured by any of these entities although clonal clusters of the closely related isolates are of particular epidemiological/clinical interest. This interest becomes even more pertinent if such drug resistance-associated and/or hypervirulent clusters demonstrate global or local population increase hence impact on the public health programs.

The *M. tuberculosis* Beijing genotype is globally spread lineage with important medical properties. The evolutionary history of the Beijing genotype is far from straightforward and was marked by some key turning points shaped by human migrations and demography. While Beijing itself likely emerged in the North of China, the ancestral lineage termed as proto-Beijing originated in the South of China [1]. Ancient or ancestral branch of the Beijing genotype is dominant in Japan, Korea, parts of China and Vietnam but extremely rare elsewhere in the world [2–6]. These strains have not been marked with particular clinically significant properties and show decreased transmission e.g., in Japan [7]. Although phylogenetic sublineages (ancient/ancestral and modern) of the Beijing genotype were first postulated in a Russian study [8], the ancient Beijing strains have been rarely found in Russia and did not attract any particular attention.

**Fig. 1:**
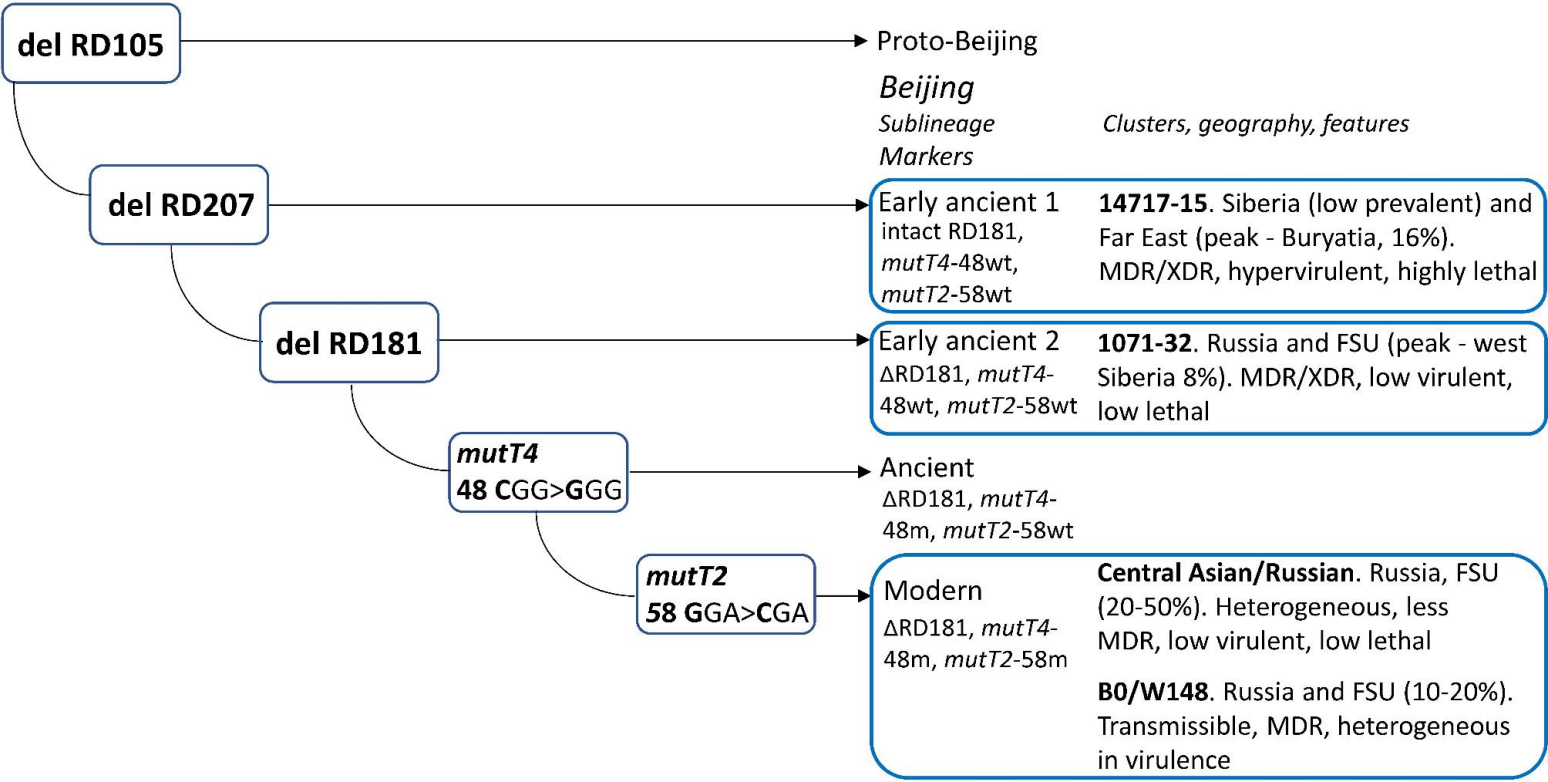
Simplified evolutionary scenario of the *M. tuberculosis* Beijing genotype, including ancient Beijing 14717-15-cluster [47]. Main Russian clusters are in bold

That being said, it was a surprise to find two clusters of exclusively MDR strains of the ancient Beijing sublineage in two locations in the Asian part of Russia [9] (see clusters 1071-32 and 14717-15 on Fig. 1). A murine model study demonstrated that one of these clusters 14717-15 that belongs to the RD181-intact sublineage, is not only MDR but highly lethal and hypervirulent [10]. In spite of this, this strain is prevalent only in one area in the Russian Far East, namely in Buryatia (16%). It was hypothesized that this situation is a result of the particular interplay of the human and bacterial genetics and long-term adaptation of these strains to the local human population.

The strain was the most lethal of all Russian Beijing strains studied to date, including the notorious Beijing B0/W148 cluster, yet its phylo- and pathogenomics and geography were not studied in sufficient detail. In the present study, we aimed, based on analysis of the expanded strain/DNA collection and whole genome sequencing, to identify pathogenetically relevant genomic features of the Beijing 14717-15 cluster, to develop a simple method of its detection and to assess its geographic distribution in Eurasia.

## Materials and methods

### Study collections

The collection included DNA extracted from *M. tuberculosis* strains obtained between 1996 and 2020, within prospective or cross-sectional studies or collected as convenience samples, characterized in our previous studies [2, 8–11]. The study was approved by the Ethics Committees of St. Petersburg Pasteur Institute, St. Petersburg, Russian Federation (protocol 41 of 14 December 2017) and the Research Institute of Phthisiopulmonology, St. Petersburg, Russian Federation (protocol 31.2 of 27 February 2017). All methods were performed in accordance with the relevant guidelines and regulations.

### Genotyping

DNA was extracted from cultured *M. tuberculosis* isolates using the CTAB-based method [12], DNA-Sorb-B kit (Interlabservis, Russia), or GenoLyse® kit (Hain Lifescience). One microliter of the DNA extracted using DNA-Sorb-B or GenoLyse® commercial kits and 10–20 ng of DNA extracted using CTAB method was used for PCR.

Spoligotyping and 24 loci MIRU-VNTR typing were performed according to standard protocols [13, 14]. The Beijing genotype was identified experimentally or *in silico* based on deletion RD207 (positions 3,120,521–3,127,920 in H37Rv genome, NC_000962.3). The main sublineages of the Beijing genotype were identified by the following molecular markers: (i) *mutT4* codon 48 CGG > GGG mutation, (ii) *mutT2* codon 58 GGA > CGA mutation, (iii) deletion RD181 (positions 2,535,429–2,536,140 in H37Rv genome, NC_000962.3). These three markers permit to differentiate between early ancient 1, early ancient 2, and classical ancient subgroups of the Beijing genotype (Fig. 1) [9]. Compared to some of the previous classifications summarized by Shitikov et al. [15], early ancient 1 and 2 correspond to Asia Ancestral 1 and 2 branches, respectively.

#### Whole genome sequencing

Whole genome sequencing was performed at the HiSeq platform (Illumina). DNA libraries were prepared using ultrasound DNA fragmentation and NEBNext Ultra DNA Library Prep Kit for Illumina (New England Biolabs). Data for the *M. tuberculosis* sequenced genomes were deposited in the NCBI Sequence Read Archive (project number PRJNA822891).

TB Profiler database (http://tbdr.lshtm.ac.uk/) was used for genotypic detection of drug resistance. MDR, pre-XDR and XDR phenotypes were defined according to the updated World Health Organization definitions: MDR are strains resistant to isoniazid and rifampicin; pre-XDR - resistant to isoniazid, rifampicin, fluoroquinolone; XDR - resistant to isoniazid, rifampicin, fluoroquinolone plus bedaquiline and/or linezolid [16].

### Bioinformatics and phylogenetic analysis

A dataset comprising *Mycobacterium tuberculosis* lineage 2 isolates with intact RD181 (*n* = 618) and one H37Rv isolate was retrieved from NCBI database (https://www.ncbi.nlm.nih.gov/sra) using SRA Toolkit v3.0.0 (https://github.com/ncbi/sra-tools) and parallel-fastq-dump v0.6.7 (https://github.com/rvalieris/parallel-fastq-dump). Quality of downloaded FASTQ files was assessed with FastQC v0.11.9 (https://github.com/s-andrews/FastQC).

These 618 genomes included 8 Russian genomes and 610 genomes from 23 other countries [1, 15, 17–21] (see Table S1 with accession numbers). The TBvar v1.1.5 workflow (https://github.com/dbespiatykh/TBvar) was used for mapping and variant calling. In brief, FASTQ reads were mapped to the reference *M. tuberculosis* H37Rv genome (RefSeq accession no. NC_000962.3) using BWA MEM v0.7.17 [22] algorithm. Mapped reads were sorted by coordinates, converted to BAM format and indexed using SAMtools v1.16.1 [23]. Subsequently, duplicate reads were removed with Sambamba v1.0 [24]. Mapping quality was assessed with SAMtools stats and mosdepth v0.3.3 [25]. All the following variant calling steps were performed with GATK4 v4.3.0.0 package [26]. All reports were aggregated with MultiQC v1.10.1 [27]. Variants effects were annotated with SIFT4G v19.0.2 [28] and SnpEff v5.1d [29].

Lineages from called SNPs were assigned with TbLG v0.1.5 (https://github.com/dbespiatykh/tblg). TB-Profiler v4.4.2 was used to discover resistance mutations and for spoligotyping [30]. To construct the phylogenies, the SNP alignment was extracted from the tab-delimited output of GATK VariantsToTable. Repetitive regions were excluded using a mask from a previously published study (available at https://github.com/mbhall88/head_to_head_pipeline/blob/master/analysis/baseline_variants/resources/compass-mask.bed) [31]. Recombinant regions from the SNP alignment were filtered out using Gubbins v3.2.1 [32]. The resulting alignment was cleaned with SNP-sites v2.5.1 [33]. Maximum likelihood (ML) phylogeny was inferred from 619 sequences with 16 220 nucleotide sites using IQ-TREE 2 v2.2.0.3 [34]. Support values were inferred from 1 000 ultrafast bootstrap replicates (UFBoot [35]) with the “-bnni” argument and from 1 000 replicates for Shimodaira-Hasegawa (SH) approximate likelihood ratio test with the “-altr” argument. Best-fit model was determined by ModelFinder [36] with the “-m MFP” argument, best-fit model according to Bayesian information criterion (BIC) was K3Pu + F + ASC + R7. *M. tuberculosis* H37Rv1998 (SRR20082811) was used as an outgroup. ML phylogeny was visualized with the ggtree v3.7.1.002 [37], ggtreeExtra v1.4.2 [38], ggplot2 v3.3.6 (https://ggplot2-book.org/), ggstar v1.0.4 (https://github.com/xiangpin/ggstar), ggplotify v0.1.0 (https://github.com/GuangchuangYu/ggplotify), ggnewscale v0.4.7 (https://github.com/eliocamp/ggnewscale), randomcoloR v1.1.0.1 (https://github.com/ronammar/randomcoloR), and tidytree v0.4.2 (https://github.com/YuLab-SMU/tidytree) packages for R v4.1.2 [39]. To construct minimum spanning tree (MST) SNP distance matrix was created using Seqtk v1.3-r106 (https://github.com/lh3/seqtk) and snp-dists v0.8.2 (https://github.com/tseemann/snp-dists). MST tree was inferred and visualized using ape v5.7 [40], igraph v1.4.1 (https://github.com/igraph/rigraph), ggnetwork v0.5.12 (https://github.com/briatte/ggnetwork), and ggplot2 v3.4.1 R packages.

The NGS data (fastq files) were used for in silico spoligotyping using SpoTyping program [41].

For the enrichment analysis Clusters of Orthologous Genes (COG) categories were annotated using eggNOG-mapper v2.1.9 [42], gene ontology (GO) categories with PANNZER tool [43] using Positive Predictive Value (PPV) cutoff of 0.5, KEGG pathways were annotated using BioServices v1.11.2 [44] Python library. Additionally, functional categories from the TubercuList database were also tested for enrichment [45]. All enrichment analyses were performed in R using Fisher’s exact test.

The significance of amino acid substitutions was assessed using PAM1 (Point Accepted Mutation 1) values calculated by PhyResSE online tool. The SIFT tool was used to predict whether an amino acid substitution affects protein function based on sequence homology and the physical properties of amino acids (https://sift.bii.a-star.edu.sg/index.html).

### PCR-RFLP analysis of Beijing 14717-15-clusters SNPs

Two SNPs at genome positions 2,423,040 and 1,448,330 were tested by *HhaI* PCR-RFLP assays.

The first SNP is at genome position 2,423,040 A > G and concerns gene *Rv2161c* (amino acid change in codon 33 Val > Ala [GTG/GCG], gene position 98T > C). This A > G mutation creates an additional site for *HhaI* (GCGC). Two primers are used for PCR of this gene region: 2423040F 5’-GTCCGGCAGCTCTCCACCG and 2423040R 5’-TGCAGTTCGTCACCGACCTGACC. PCR conditions: 95 °C, 5 min; 35 cycles of 95 °C, 30 s, 67 °C, 20 s, 72 °C, 20 s, and final extension 72 °C, 3 min. PCR product size was 146 bp. After *HhaI* digestion at 37 °C for 3 h, the fragments were separated in 1.4% standard agarose gel. The profile for wild type allele consists of two fragments 87 and 59 bp, and in case of mutation, of three fragments 65, 22, and 59 bp.

The second SNP is at genome position 1,448,330 G > T and concerns gene *Rv1293* (*lysA*) (silent mutation in codon 101-Ala). This mutation inactivates the single *HhaI* site in this gene fragment. Two primers are used for PCR of this gene region: 1448330F 5’-TGGAAGTGGGGCGAACGTGC and 1448330R 5’-TTGACCGCAGCGGTCAACTCTGA. PCR conditions were the same as above, and PCR product size was 201 bp. After *HhaI* digestion at 37 °C for 3 h, the fragments were separated in 1.4% standard agarose gel. The profile for wild type allele consists of two fragments 121 and 80 bp, and in case of mutation, the PCR product remains undigested 201 bp.

## Results and discussion

### Phylogenomic position of Beijing 14717-15-cluster

**Fig. 2:**
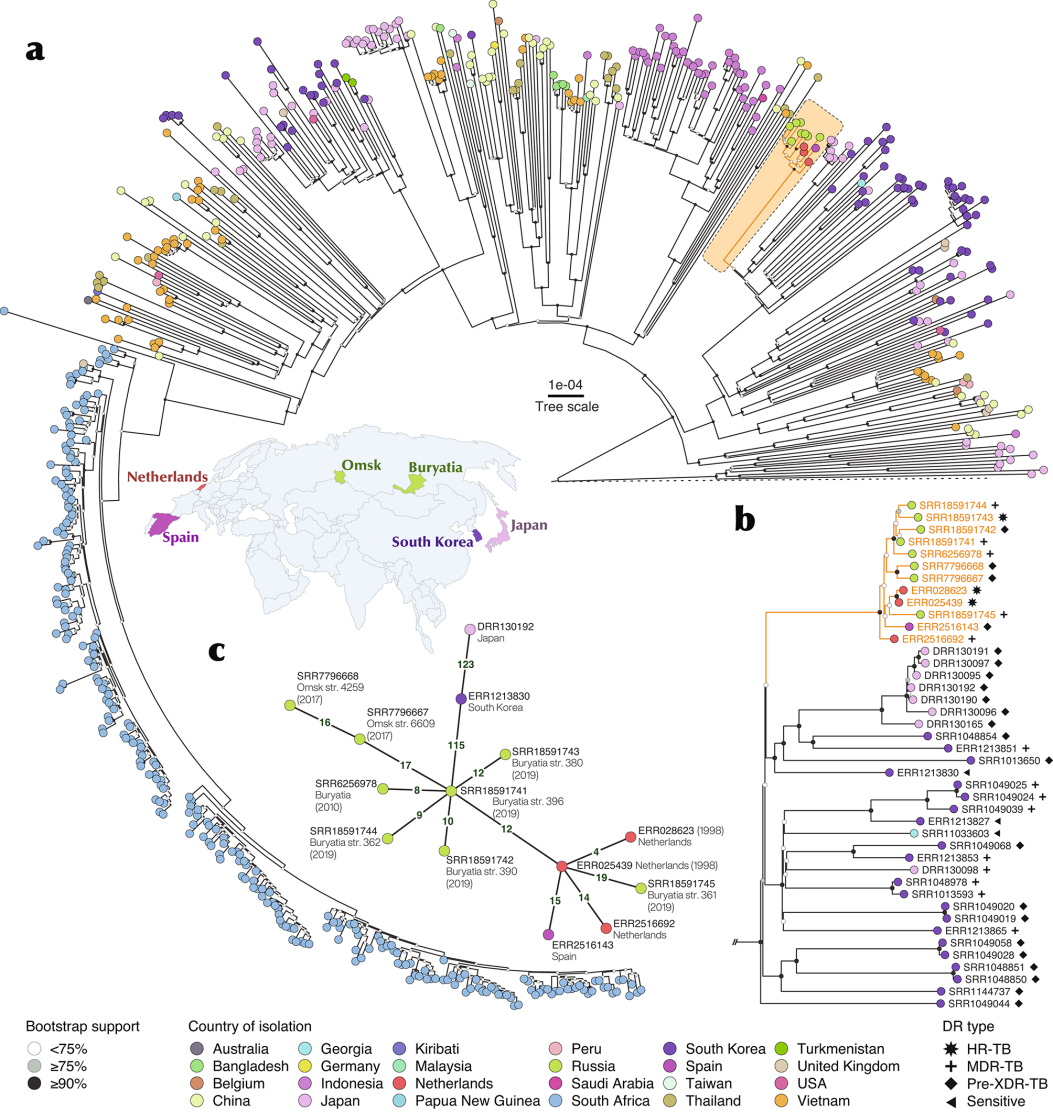
Phylogenomic analysis of the Beijing genotype isolates with intact RD181. **(A)** Global dataset (n = 618); **(B)** Beijing 14717-15-cluster and neighboring branches; **(C)** Minimum spanning tree of the Beijing 14717-15-cluster with information on the region of origin of Russian strains and year of isolation of the isolates (when available)

Phylogenomic analysis of the Beijing isolates with intact RD181 (early ancient 1 sublineage of the Beijing genotype) was performed on 8 Russian genomes (2 from Omsk, West Siberia and 6 from Buryatia, Far East) and 610 genomes from 23 countries, mostly from East and Southeast Asia (Table S1, Fig. 2a). All Russian isolates clustered in a separate branch on the tree (see the uppermost branch on Fig. 2b). Four isolates from Europe were also found within this cluster and included three from the Netherlands and one from Spain. VNTR typing of the Russian isolates assigned all of them to the Mlva type 14717-15 and related profiles. For this reason, we term this branch the Beijing 14717-15-cluster. All isolates of this cluster had *in silico* deduced spoligotype SIT269 (Table S2) that is a derived profile from the classical Beijing SIT1 by deletion of spacers 35 and 36. The experimental spoligotyping profiles were available for the Russian isolates and were concordant with their *in silico* spoligoprofiles.

On the phylogenetic tree, the relatively nearest neighbors of the Beijing 14717-15-cluster were isolates from Korea and Japan (Fig. 2b). All Beijing 14717-15-cluster isolates and some Korean isolates had spoligotype SIT269 however, given a limited number of spacers in the CRISPR locus of the Beijing genotype, this profile may be a result of convergent evolution and does not necessarily indicate a common origin.

The phylogenetic network (Fig. 2c) shows that 115 SNPs separated Russian cluster from the most recent common ancestor with isolates from Korea which implies only very distant relation of these isolates. It may be noted that Korean isolates were separated by even more SNPs between them (mostly 130–200 SNPs [not shown]) that likely correlates with their long-term evolution in a country of the endemic high prevalence of the RD181-intact ancient Beijing sublineage, i.e. Korea.

Resistance mutations were detected *in silico* based on the WGS data (Fig. 2b, Table S3). One should keep in mind a certain bias of the studied isolates from some countries, in particular a collection from Korea included mainly drug resistant isolates. On the other hand, all Russian isolates came from the population-based studies and were not preselected in any way. In this view, MDR/pre-XDR status of Russian isolates is noteworthy. All isolates of this cluster harbored two-mutation signature of the high-confidence resistance mutations *katG* Ser315Thr and *rpsL* Lys88Arg [46]. Interestingly, two isolates from the Netherlands, 1998, harbored only two first-line drugs resistance mutations (in *rpsL88* and *katG315)* and thus were likely brought to the Netherlands during the early dissemination of this strain. *katG* Ser315Thr is the most frequent INH-resistance associated mutation and its presence is expected. The other mutation *rpsL* Lys88Arg is also a well-known high-confidence mutation associated with STR resistance but it is less frequent than *rpsL43* mutation [46]. Together these two mutations *katG* Ser315Thr and *rpsL* Lys88Arg may be considered as a characteristic marker of this cluster although they alone cannot be used for its identification.

We further identified polymorphisms specific of the Beijing 14717-15-cluster (Table S4). They included 55 SNPs in CDS (35 non-synonymous, 20 synonymous) and 10 SNPs in intergenic regions. Some of the SNPs were in the genes related to mycobacterial virulence and adaptation and could hypothetically influence an increased virulence and lethality of this cluster which was demonstrated previously in both murine model and in TB patients [10, 47]. For example, *PPE18* is known to be related to immune evasion [48–50]. Some other genes (*fadE17*, *mmpS3*, *pks7*) are related to adaptation and virulence [51, 52]. Nevertheless, gene function enrichment analysis revealed that the genes with nonsynonymous mutations were only enriched in lipid metabolism category according to Tuberculist.

Based on the PAM 1 values, it is possible to hypothesize a significant influence of the amino acid change and such SNPs with PAM1 below 5 were identified in 10 genes including *pks7*, *fadE17*, *hpx* (Table S4).

In addition, SIFT *P* values were calculated for 35 nonsynonymous mutations. As a result, 12 SNPs in genes of different categories (Lipid metabolism, Regulatory proteins, Intermediary metabolism and respiration, PE/PPE, Cell wall and cell processes) were found to significantly affect protein function (P < 0.05) (Table 1). Information on these 12 genes was searched in Pubmed but only few of them were found and without relation to pathobiological properties. However, at least some of these genes such as, polyketide synthase Pks7, methyltransferase Rv0567, conserved transmembrane protein Rv0064, transcriptional regulatory protein Rv0823c and two PE/PPE genes deserve particular attention. In particular, *Pks* genes encoding the polyketide synthases are involved in the lipopolysaccharide and complex lipids biosynthesis [53]. Mutations in the *pks* genes were also suggested to have a compensatory role in drug resistance [51, 52].

**Table 1:** Twelve *in silico* predicted significant mutations characteristic of the Beijing 14717-15-cluster

Gene	AA exchange	PAM1	SIFT *P*	Product	Function, category (https://mycobrowser.epfl.ch/)
*Rv0360c*	Trp89STOP	0	-	Conserved protein	Conserved hypotheticals. Function unknown
*Rv1866*	Asp699Tyr	0	0.01	Conserved protein	Lipid metabolism. Function unknown, but supposed involvement in lipid degradation.
*Rv1661 (pks7)*	Leu2076Arg	1	0.01	Probable polyketide synthase Pks7	Lipid metabolism. Potentially involved in some intermediate steps for synthesis of polyketide molecule which may be involved in secondary metabolism
*Rv1154c*	Met(s)57Ile	2	0.00	Hypothetical protein	Conserved hypotheticals. Function unknown
*Rv0567*	Ser76Cys	5	0.02	Probable methyltransferase/methylase	Intermediary metabolism and respiration. Causes methylation.
*Rv0064*	Ala225Pro	13	0.01	Probable conserved transmembrane protein	Cell wall and cell processes.
*Rv2263*	Gly25Ser	16	0.00	Possible oxidoreductase	Intermediary metabolism and respiration. Oxidoreduction.
*Rv3350c (PPE56)*	Gly2482Ser	16	0.00	PPE family protein	Function unknown
*Rv1077 (cbs)*	Val77Ala	18	0.02	Probable cystathionine beta-synthase	Intermediary metabolism and respiration. Thought to be involved in homocysteine transulfuration.
*Rv0823c*	Thr218Met(s)	32	0.00	Possible transcriptional regulatory protein	Regulatory proteins. Thought to be involved in transcriptional mechanism.
*Rv0152c (PE2)*	Asp217Asn	36	0.02	PE family protein	Function unknown
*Rv2607 (pdxH)*	Asp84Asn	36	0.04	Probable pyridoxamine 5’-phosphate oxidase	Intermediary metabolism and respiration. Involved in biosynthesis of pyridoxine (vitamin B6) and pyridoxal phosphate.

Substitution is predicted to affect protein function if SIFT P < 0.05. Database UniProt + TrEMBL was used SIFT analysis

### PCR-RFLP assay for detection of Beijing 14717-15-cluster

Among cluster-specific SNPs identified above, we selected two functionally neutral SNPs (PAM1 = 9867) and designed PCR-RFLP assays to detect them. These SNPs were in genome positions 1,448,330 G > T (Rv1293 Ala101Ala) and 2,423,040 A > G (Rv2161c Val(s)33Ala). The neutral SNPs reflect a neutral evolution non-influenced by selection pressure and unlikely to independently occur in different and unrelated phylogenetic groups. The use of two SNPs enhances the reliability of detection of this cluster.

**Fig. 3:**
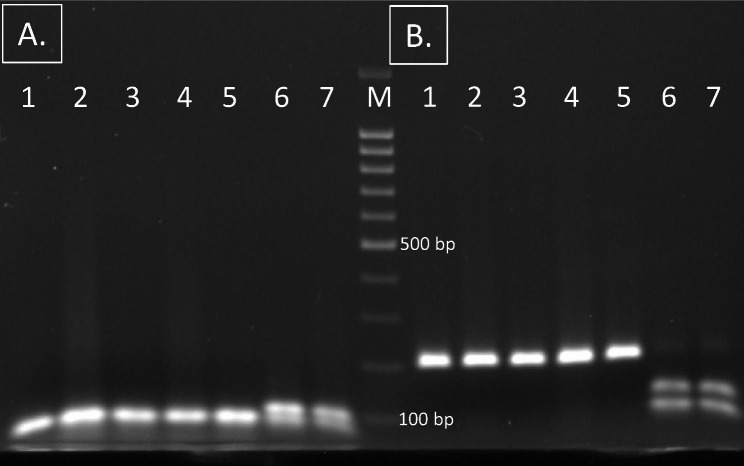
PCR *HhaI*-RFLP detection of Beijing 14717-15-cluster based on: **(A)** SNP at 2,423,040 A > G (*Rv2161c* Val33Ala) and **(B)** SNP at 1,448,330 G > T (*Rv1293* Ala101Ala). Lanes 1–5 – Beijing 14717-15-cluster. Lanes 6–7 – other genotypes. М – molecular weight marker 100 bp ladder (Fermentas). The raw gel image is shown in Figure S1

Both SNPs can be detected by *HhaI*-RFLP analysis of the amplified PCR regions (Fig. 3). PCR conditions are the same for both genes, and both PCR products are digested (separately) by the same *HhaI* endonuclease. Both PCR-RFLP assays were optimized with isolates with known WGS sequences and VNTR profiles. Both mutations were found only in isolates of the Beijing 14717-15 cluster.

These two phylogenetic SNPs of the Beijing 14717-15 cluster were screened for specificity in the proprietary Beijing global genome databases (> 6000 genomes [Dr. Joao Perdigao, Universidade de Lisboa, Portugal, and > 10,000 genomes [Dr Egor Shitikov, Lopukhin Federal Research and Clinical Center of Physical-Chemical Medicine, Russia]). As a result, these two SNPs were found robustly specific and unique for the Beijing 14717-15-cluster isolates.

A strategy to target a limited number of SNPs (at least two) was recommended and applied to identify specific strains or clones by PCR based assays [54–56] which increases the robustness. Thus, analysis of both targeted SNPs is the most robust method to detect the Beijing 14717-15-cluster. Nonetheless, detection of particular clusters/genotypes based on use of a single marker is an acceptable and parsimonious approach, provided that such marker was proven specific and sensitive in the validation studies and this concerns both detection of the particular clusters and families and the development of the SNP-barcode system [15, 57]. In this view, since analysis of the two SNPs showed completely concordant results, testing of any of them appears the most practical and time-saving approach to trace this clinically significant MDR Beijing 14717-15-cluster.

It should be noted that the strains with the intact RD181 locus belong to the early ancient sublineage of the Beijing genotype, which is very heterogeneous and includes strains with diverse VNTR profiles. In this sense, the SNPs identified by us are markers only of the Beijing cluster specific to Russia (primarily Buryatia), but not markers of the entire heterogeneous RD181-intact branch within deeply-rooted ancestral Beijing sublineage.

### Geographic screening of Beijing 14717-15-cluster

The two PCR-RFLP assays were further applied to the Beijing genotype isolates that represented different Beijing sublineages and had different VNTR profiles. These validation collections included isolates from Europe, Russia, Central and East Asia. The PCR-RFLP analysis of two SNPs correctly assigned all isolates with known Mlva 14717-15 and related profiles to the Beijing 14717-15-cluster. The method has 100% sensitivity and 100% specificity to detect Beijing 14717-15-cluster.

**Fig. 4:**
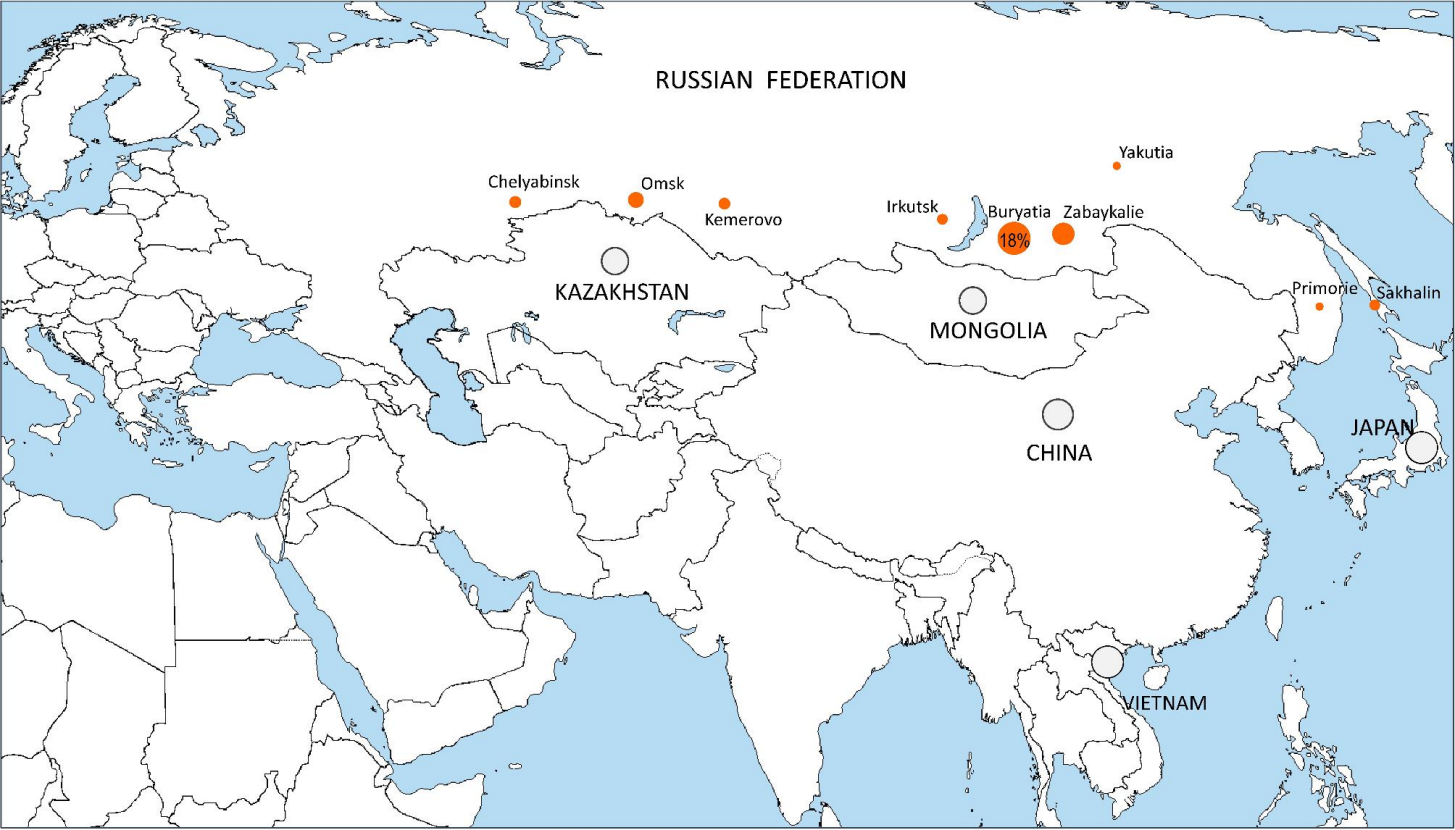
Geographic distribution of the Beijing 14717-15-cluster isolates in Russian regions, based on results of this study and previous publication [10, 57–59]. Circle size is roughly proportional to the percent of these isolates in the local *M. tuberculosis* population. Gray shade in the circle means absence of these isolates in the analyzed collection (irrespective of the circle size)

We further applied these PCR-RFLP assays to screen the available DNA collections from Russian regions and other countries. Results summarizing the above validation and screening analysis are shown in Table 2; Fig. 4 and demonstrate the clear peak of the Beijing 14717-15-cluster in Buryatia, Far East.

**Table 2:** Detection of Beijing 14717-15 in retrospective local collections

Country, region	Total	Beijing genotype	Beijing 14717-15 cluster, number and % in total local collection
Russia, Western Siberia, Omsk	482	321	12 (2.5%)
Russia, Eastern Siberia, Irkutsk	393	239	8 (2%)
Russia, Far East, Buryatia	499	342	89 (18%)
Russia, Far East, Zabaykalsky krai	62	41	5 (8%)
Russia, Far East, Yakutia	377	165	3 (0,8%)
Russia, Far East, Primorsky krai	97	68	1 (1%)
Russia, Northwest (Komi, Karelia, Kaliningrad)	371	184	0
Belarus	93	48	0
Estonia			
Greece	19	19	0
Albania	5	5	0
Bulgaria	93	0	0
Kazakhstan	148	103	0
China, Beijing	74	45	0
Vietnam, Hanoi and Ho Chi Minh	53	37	0
Japan, Okinawa	71	71	0
Mongolia	147	105	0

Note. The Beijing genotype was determined based on spoligotyping or VNTR typing. Strains of Beijing 14717-15 cluster were determined based on SNP testing

Analysis of the available archival strains isolated in 1996–2002 in northwestern Russia (St. Petersburg and other regions) did not reveal the isolates of the Beijing 14717-15-cluster. However, two isolates of this cluster were detected in the Netherlands in 1998. One possibly related isolate (based on 12-MIRU-VNTR typing) was described in Lithuania, and was isolated in 2007 [9].

We additionally looked at the geographic distribution of the main Russian clusters of the modern sublineage of the Beijing genotype (B0/W148 and Central Asian Russian) and two main clusters of the ancient sublineage (Beijing 14717-15 and Beijing 1071-32), based on results of this study and previous publication [9, 58–60]. This comparison showed the overall prevalence of the modern Beijing clusters across Russia and presence of Beijing 1071-32 at low prevalence but also indifferent parts of European Russia and Western Siberia. In this view, the high 18% prevalence of the Beijing 14717-15-cluster in Buryatia is in the striking contrast with its almost complete absence elsewhere.

We note that percent of this cluster roughly correlates with proportion of the Buryat ethnic group in Buryatia itself and its neighbors. Due to human influx from European and Siberian parts of Russia since the 1930s, the proportion of Buryats decreased from 44% to 1926 to 19% in 1970 but remains stable in the last 20–30 years and makes up to 28–30% of the total population of this region (https://en.wikipedia.org/wiki/Buryatia#Demographics). Buryats also live in the neighboring provinces in Far East and Siberia. Thus, presently, the percent of Buryats in Buryatia is 30%, in Zabaykalie 8% and in Irkutsk 3%. In turn, percent of the Beijing 14717-15-cluster in these areas is 18%, 8%, and 2%, respectively. We do not have information on the ethnic background on the patients in the previous studies but the above figures are suggestive of some correlation.

Interestingly, no Beijing 14717-15 strains were found in the neighboring Mongolia [59]. Buryat and Mongol languages are related, and Y chromosome and mtDNA based study identified common genetic components for Buryats and Mongols [61, 62] but Buryats were separated form Mongols very long ago, and definitely long before emergence of this particular *M. tuberculosis* strain. The noticeable decrease in frequency of N1c1 haplogroup in western direction and the presence of a significant proportion of unique haplotypes in Buryats indicate the absence of the intensive gene drift from Buryats to Mongols [61]. Based on mtDNA graphs, Buryats are very heterogeneous and only one of their subgroups is close to Mongols [62]. A relatively mass migration of Buryat people to Mongolia took place 90 years ago when they flew Red Army. However, no significant human movement took place since the 1930s and the two countries are separated by the state borders. This could be the reason why this strain was not brought to Mongolia form the neighboring Buryatia.

## Conclusions

Important strains may unexpectedly emerge among minor genotype lineages as was shown for genotypes of the Euro-American lineage, such as drug-resistant clones within Haarlem, LAM, Ural, NEW-1 families [63–66]. Herein described the Beijing 14717-15-cluster is the other relevant example. The strain was shown concordantly lethal and virulent in mice and human studies [10, 47]. Its elevated prevalence only in one region was linked to some hypothetical interplay of human immune system and the genetic background of this strain during local coevolution and long-term coadaptation. Further studies including GWAS-based may eventually shed more light.

Cluster-specific SNPs that significantly affect protein function were identified in 12 genes of different categories (Lipid metabolism, Regulatory proteins, Intermediary metabolism and respiration, PE/PPE, Cell wall and cell processes). Most of these genes were previously unreported and could potentially be associated with increased pathogenic properties of these strains.

Furthermore, when the entire bacterial genome is considered, not only SNPs but also insertions and deletions could be cluster-specific and functionally significant. A further study of such alterations and their association with pathogenic properties of the isolates is warranted through more complete genome sequencing (including de-novo assembly and long-read sequencing), and experimental allelic exchange approach.

The Russian isolates of the cluster 14717-15 were from the Asian part of the country. They had two common resistance mutations *rpsL* Lys88Arg and *katG* Ser315Thr. Phylogenetically, their neighbors were isolates from Korea, while the Russian isolates from both Omsk and Buryatia and some of the Korean isolates had a characteristic spoligoprofile SIT269 (derived from the classic spoligo profile Beijing - SIT1). However, the distance between Russian and the closest Korean isolates was at least 115 SNPs (corresponding to ~ 230 years, based on generally assumed mutation rate of 0.5 SNPs/genome/year) and SIT269 may well result from convergent evolution. In this view, the hypothesis of the Korean distant descent of this medically significant Russian cluster remains a speculation. Availability of more genomes from East Asia should hopefully permit more robust reconstruction of its evolutionary history while omics studies may help to reach a more informed view on pathobiological relevance of its genetic variation.

## Electronic supplementary material

Below is the link to the electronic supplementary material.

Supplementary Material 1

Supplementary Material 2

## Data Availability

The data that support the findings of this study are presented in Supplementary Table S1 that provides a complete list of accession numbers for all genomes used in this study. In particular, the genomes sequenced in our laboratory were deposited in NCBI Short Read Archive under accession numbers: SRR18591745, SRR18591744, SRR18591743, SRR18591742, SRR18591741, SRR7796667, SRR7796668.

## References

[1] Luo T, Comas I, Luo D (2015). Southern East Asian origin and coexpansion of Mycobacterium tuberculosis Beijing family with Han Chinese. Proc Natl Acad Sci USA.

[2] Yin QQ, Liu HC, Jiao WW (2016). Evolutionary history and ongoing transmission of phylogenetic sublineages of Mycobacterium tuberculosis Beijing genotype in China. Sci Rep.

[3] Shamputa IC, Lee J, Allix-Béguec C (2010). Genetic diversity of Mycobacterium tuberculosis isolates from a tertiary care tuberculosis hospital in South Korea. J Clin Microbiol.

[4] Maeda S, Hijikata M, Hang NTL (2019). Genotyping of Mycobacterium tuberculosis spreading in Hanoi, Vietnam using conventional and whole genome sequencing methods. Infect Genet Evol.

[5] Kang HY, Wada T, Iwamoto T (2010). Phylogeographical particularity of the Mycobacterium tuberculosis Beijing family in South Korea based on international comparison with surrounding countries. J Med Microbiol.

[6] Maeda S, Hang NT, Lien LT (2014). Mycobacterium tuberculosis strains spreading in Hanoi, Vietnam: Beijing sublineages, genotypes, drug susceptibility patterns, and host factors. Tuberculosis (Edinb).

[7] Iwamoto T, Fujiyama R, Yoshida S (2009). Population structure dynamics of Mycobacterium tuberculosis Beijing strains during past decades in Japan. J Clin Microbiol.

[8] Mokrousov I, Narvskaya O, Otten T (2002). Phylogenetic reconstruction within Mycobacterium tuberculosis Beijing genotype in northwestern Russia. Res Microbiol.

[9] Mokrousov I, Vyazovaya A, Pasechnik O (2019). Early ancient sublineages of Mycobacterium tuberculosis Beijing genotype: unexpected clues from phylogenomics of the pathogen and human history. Clin Microbiol Infect.

[10] Vinogradova T, Dogonadze M, Zabolotnykh N (2021). Extremely lethal and hypervirulent Mycobacterium tuberculosis strain cluster emerging in Far East, Russia. Emerg Microbes Infect.

[11] Mokrousov I, Vyazovaya A, Sinkov V, Gerasimova A, Ioannidis P, Jiao W, Khromova P, Papaventsis D, Pasechnik O, Perdigão J, Rastogi N, Shen A, Skiba Y, Solovieva N, Suffys P, Tafaj S, Umpeleva T, Vakhrusheva D, Yarusova I, Zhdanova S, Zhuravlev V, Ogarkov O (2021). Practical approach to detection and surveillance of emerging highly resistant Mycobacterium tuberculosis Beijing 1071-32-cluster. Sci Rep.

[12] van Embden JD, Cave MD, Crawford JT (1993). Strain identification of Mycobacterium tuberculosis by DNA fingerprinting: recommendations for a standardized methodology. J Clin Microbiol.

[13] Mokrousov I, Rastogi N (2015). Spacer-based Macroarrays for CRISPR genotyping. Methods Mol Biol.

[14] Supply P, Allix C, Lesjan S (2006). Proposal for standardization of optimized mycobacterial interspersed repetitive unit-variable-number tandem repeat typing of Mycobacterium tuberculosis. J Clin Microbiol.

[15] Shitikov E, Kolchenko S, Mokrousov I (2017). Evolutionary pathway analysis and unified classification of east asian lineage of Mycobacterium tuberculosis. Sci Rep.

[16] Roelens M, Battista Migliori G, Rozanova L (2021). Evidence-based definition for extensively drug-resistant tuberculosis. Am J Respir Crit Care Med.

[17] Thawornwattana Y, Mahasirimongkol S, Yanai H (2021). Revised nomenclature and SNP barcode for Mycobacterium tuberculosis lineage 2. Microb Genom.

[18] Napier G, Campino S, Merid Y (2020). Robust barcoding and identification of Mycobacterium tuberculosis lineages for epidemiological and clinical studies. Genome Med.

[19] Klopper M, Heupink TH, Hill-Cawthorne G (2020). A landscape of genomic alterations at the root of a near-untreatable tuberculosis epidemic. BMC Med.

[20] Bespiatykh D, Bespyatykh J, Mokrousov I (2021). A Comprehensive Map of Mycobacterium tuberculosis Complex Regions of Difference. mSphere.

[21] Mokrousov I, Sinkov V, Vyazovaya A (2020). Genomic signatures of drug resistance in highly resistant Mycobacterium tuberculosis strains of the early ancient sublineage of Beijing genotype in Russia. Int J Antimicrob Agents.

[22] Li H, Durbin R (2009). Fast and accurate short read alignment with Burrows–Wheeler transform. Bioinformatics.

[23] Danecek P, Bonfield JK, Liddle J (2021). Twelve years of SAMtools and BCFtools. Gigascience.

[24] Tarasov A, Vilella AJ, Cuppen E (2015). Sambamba: fast processing of NGS alignment formats. Bioinformatics.

[25] Pedersen BS, Quinlan AR (2018). Mosdepth: quick coverage calculation for genomes and exomes. Bioinformatics.

[26] Depristo MA, Banks E, Poplin R (2011). A framework for variation discovery and genotyping using next-generation DNA sequencing data. Nat Genet.

[27] Ewels P, Magnusson M, Lundin S (2016). MultiQC: summarize analysis results for multiple tools and samples in a single report. Bioinformatics.

[28] Vaser R, Adusumalli S, Leng SN (2016). SIFT missense predictions for genomes. Nat Protoc.

[29] Cingolani P, Platts A, Wang LL (2012). A program for annotating and predicting the effects of single nucleotide polymorphisms, SnpEff: SNPs in the genome of Drosophila melanogaster strain w1118; iso-2; iso-3. Fly (Austin).

[30] Phelan JE, O’Sullivan DM, Machado D (2019). Integrating informatics tools and portable sequencing technology for rapid detection of resistance to anti-tuberculous drugs. Genome Med.

[31] Hall MB, Rabodoarivelo MS, Koch A (2023). Evaluation of Nanopore sequencing for Mycobacterium tuberculosis drug susceptibility testing and outbreak investigation: a genomic analysis. Lancet Microbe.

[32] Croucher NJ, Page AJ, Connor TR (2015). Rapid phylogenetic analysis of large samples of recombinant bacterial whole genome sequences using Gubbins. Nucleic Acids Res.

[33] Page AJ, Taylor B, Delaney AJ (2016). SNP-sites: rapid efficient extraction of SNPs from multi-FASTA alignments. Microb Genom.

[34] Minh BQ, Schmidt HA, Chernomor O (2020). IQ-TREE 2: New Models and efficient methods for phylogenetic inference in the genomic era. Mol Biol Evol.

[35] Hoang DT, Chernomor O, von Haeseler A (2018). UFBoot2: improving the Ultrafast bootstrap approximation. Mol Biol Evol.

[36] Kalyaanamoorthy S, Minh BQ, Wong TKF (2017). ModelFinder: fast model selection for accurate phylogenetic estimates. Nat Methods.

[37] Xu S, Li L, Luo X (2022). Ggtree: a serialized data object for visualization of a phylogenetic tree and annotation data. iMeta.

[38] Xu S, Dai Z, Guo P (2021). ggtreeExtra: Compact visualization of richly annotated phylogenetic data. Mol Biol Evol.

[39] R Core Team. R: A Language and Environment for Statistical Computing. 2021.

[40] Paradis E, Claude J, Strimmer K (2004). APE: analyses of Phylogenetics and Evolution in R language. Bioinformatics.

[41] Xia E, Teo YY, Ong RT (2016). SpoTyping: fast and accurate in silico Mycobacterium spoligotyping from sequence reads. Genome Med.

[42] Cantalapiedra CP, Hernandez-Plaza A, Letunic I (2021). eggNOG-mapper v2: functional annotation, Orthology assignments, and Domain Prediction at the Metagenomic Scale. Mol Biol Evol.

[43] Törönen P, Holm L (2022). PANNZER—A practical tool for protein function prediction. Protein Sci.

[44] Cokelaer T, Pultz D, Harder LM (2013). BioServices: a common Python package to access biological web services programmatically. Bioinformatics.

[45] Lew JM, Kapopoulou A, Jones LM (2011). TubercuList – 10 years after. Tuberculosis.

[46] World Health Organisation (2018). The use of next-generation sequencing technologies for detection of mutations associated with drug resistance in Mycobacterium tuberculosis complex: technical guide.

[47] Mokrousov I, Pasechnik O, Vyazovaya A (2022). Impact of pathobiological diversity of Mycobacterium tuberculosis on clinical features and lethal outcome of tuberculosis. BMC Microbiol.

[48] Hakim JMC, Yang Z (2021). Predicted structural variability of Mycobacterium tuberculosis PPE18 protein with immunological implications among clinical strains. Front Microbiol.

[49] Dolasia K, Nazar F, Mukhopadhyay S (2021). Mycobacterium tuberculosis PPE18 protein inhibits MHC class II antigen presentation and B cell response in mice. Eur J Immunol.

[50] Tantivitayakul P, Ruangchai W, Juthayothin T (2020). Homoplastic single nucleotide polymorphisms contributed to phenotypic diversity in Mycobacterium tuberculosis. Sci Rep.

[51] Zhang H, Li D, Zhao L (2013). Genome sequencing of 161 Mycobacterium tuberculosis isolates from China identifies genes and intergenic regions associated with drug resistance. Nat Genet.

[52] Kuan CS, Chan CL, Yew SM (2015). Genome analysis of the First Extensively Drug-Resistant (XDR) Mycobacterium tuberculosis in Malaysia provides insights into the genetic basis of its Biology and Drug Resistance. PLoS ONE.

[53] Domenech P, Reed MB, Barry CE (2005). Contribution of the Mycobacterium tuberculosis MmpL protein family to virulence and drug resistance. Infect Immun.

[54] Pérez-Lago L, Martínez-Lirola M, García S (2016). Urgent implementation in a hospital setting of a strategy to rule out secondary cases caused by Imported extensively drug-resistant Mycobacterium tuberculosis strains at diagnosis. J Clin Microbiol.

[55] Genestet C, Perdigão J, Herranz M (2021). Expanded tracking of a Beijing Mycobacterium tuberculosis strain involved in an outbreak in France. Travel Med Infect Dis.

[56] Stucki D, Ballif M, Bodmer T (2015). Tracking a tuberculosis outbreak over 21 years: strain-specific single-nucleotide polymorphism typing combined with targeted whole-genome sequencing. J Infect Dis.

[57] Millán-Lou MI, Alonso H, Gavin P (2012). Rapid test for identification of a highly transmissible Mycobacterium tuberculosis Beijing strain of sub-saharan origin. J Clin Microbiol.

[58] Zhdanova SN, Ogarkov OB, Savilov ED (2019). Molecular epidemiology of tuberculosis in northern Asia and its manifestations against the background of the HIV epidemic.

[59] Zhdanova S, Mokrousov I, Orlova E (2022). Transborder molecular analysis of drug-resistant tuberculosis in Mongolia and Eastern Siberia, Russia. Transbound Emerg Dis.

[60] Badleyeva MV, Zhdanova SN, Baasansuren E et al. Molecular-Genetic Features of Tuberculosis in Mongolia and in Russian Bordering Regions/ Epidemiologiya vaccinoprofilaktika. 2017 (5) 53–57. In Russian.

[61] Kharkov VN, Khamina KV, Medvedeva OF (2014). Gene pool of Buryats: clinal variability and territorial subdivision based on data of Y-chromosome markers. Russ J Genet.

[62] Gibert M, Theves C, Ricaut FX (2010). mtDNA variation in the buryat population of the Barguzin Valley: new insights into the micro-evolutionary history of the Baikal area. Ann Hum Biol.

[63] Mardassi H, Namouchi A, Haltiti R (2005). Tuberculosis due to resistant Haarlem strain, Tunisia. Emerg Infect Dis.

[64] Skhairia MA, Dekhil N, Mhenni B (2021). Successful expansion of Mycobacterium tuberculosis Latin American and Mediterranean sublineage (L4.3/LAM) in Tunisia mainly driven by a single, long-established clonal complex. Int J Infect Dis.

[65] Sinkov V, Ogarkov O, Mokrousov I (2018). New epidemic cluster of pre-extensively drug resistant isolates of Mycobacterium tuberculosis Ural family emerging in Eastern Europe. BMC Genomics.

[66] Mokrousov I (2016). Emerging resistant clone of Mycobacterium tuberculosis in west Asia. Lancet Infect Dis.

