# Supplementary material for: Insight into pathogenomics and phylogeography of hypervirulent and highly-lethal Mycobacterium tuberculosis strain cluster
Source: BMC Infect Dis. 2023 Jun 23;23:426. doi: 10.1186/s12879-023-08413-7 (PMC10288800; doi:10.1186/s12879-023-08413-7)
Supplement: Supplementary file 1 — Supplementary Material 1 [file 12879_2023_8413_MOESM1_ESM.pdf]

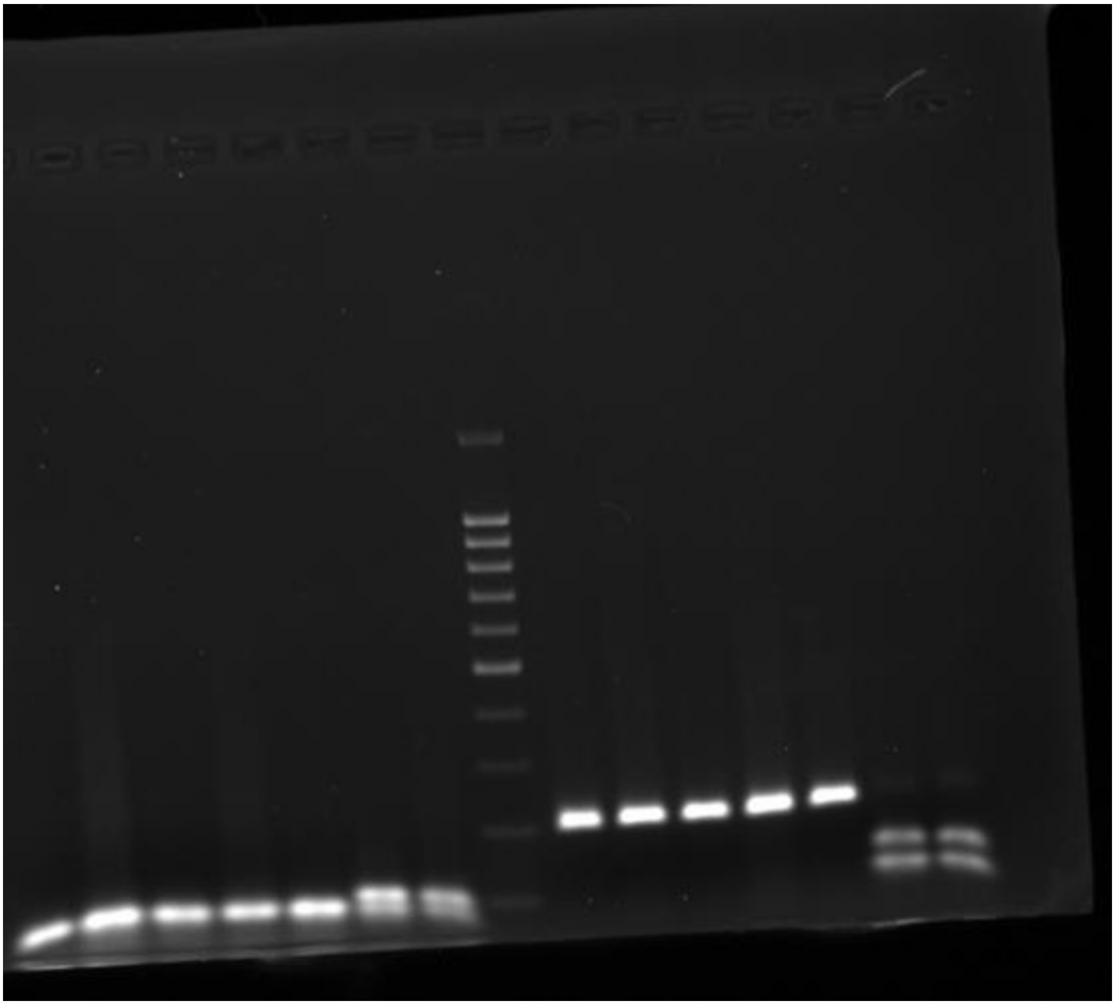

Figure S1. Supporting figure for Figure 3. PCR HhaI-RFLP detection of Beijing 14717-15-cluster based on: (A) SNP at 2423040 A>G (*Rv2161c* Val(s)33Ala) and (B) SNP at 1448330 G>T (*Rv1293* Ala101Ala). Lanes 1-5 – Beijing 14717-15-cluster. Lanes 6-7 – other genotypes. M – molecular weight marker 100 bp ladder (Fermentas).

Note. The image with gel lanes shown in Figure 2 shows examples of different prfiles for wild or mutant alleles of the targeted SNPs. This image was not changed or cropped in any way. The supporting Figure S3 is shown solely to formally address BMC digital image integrity policies.
